# Supplementary material for: Regional Differences in COVID-19 Vaccine Hesitancy in December 2020: A Natural Experiment in the French Working-Age Population
Source: Vaccines (Basel). 2021 Nov 20;9(11):1364. doi: 10.3390/vaccines9111364 (PMC8622681; doi:10.3390/vaccines9111364)
Supplement: Supplementary file 1 [file vaccines-09-01364-s001.zip › vaccines-1347962-supplementary.pdf]

Regional Differences in COVID-19 Vaccine Hesitancy in December 2020: A Natural Experiment in the French Working-Age Population

Table S1. Choice tasks and overall responses (December 2020, France) 2

Table S2. Prevalence of detected SARS-CoV-2 infection in the French working age population 4

Table S3. Characteristics of participants without prior SARS-CoV-2 infection by region of residency (December 2020, France) 5

Table S4. Outright refusal of COVID-19 vaccination (December 2020, France) 10

Figure S1. Acceptance of Pfizer or Moderna vaccines predicted in the working age population (December 2020, France) 12

Figure S2. Acceptance of AstraZeneca vaccine predicted in the working age population (December 2020, France) 13

Table S1. Choice tasks and overall responses (December 2020, France).

Block	Choice task	Vaccine	Vaccine efficacy	Vaccine immunity duration	Vaccine safety	Vaccine manufacturer	Place to be vaccinated	Vaccine uptake (*n* = 4415)	Vaccine uptake without outright vaccination refusal (*n* = 2592)
Block A (*n* = 2207)	1	A	90%	No booster needed	Unknown risk	European Union	Your general practice	556 (25.2)	556 (42.8)
		B	100%	Booster every 6 months	1/100,000	China	Your local pharmacy	375 (17)	375 (28.9)
	2	A	90%	Booster every year	1/10,000	European Union	Mass vaccination centre	694 (31.4)	694 (53.5)
		B	80%	No booster needed	Unknown risk	United States of America	Your general practice	247 (11.2)	247 (19)
	3	A	100%	Booster every year	1/10,000	United States of America	Your local pharmacy	664 (30.1)	664 (51.2)
		B	50%	Booster every 6 months	1/1,000,000	China	Mass vaccination centre	172 (7.8)	172 (13.3)
	4	A	50%	Booster every year	1/1,000,000	China	Your general practice	325 (14.7)	325 (25)
		B	80%	Booster every 6 months	Unknown risk	United States of America	Your local pharmacy	421 (19.1)	421 (32.4)
	5	A	80%	Booster every year	1/100,000	United States of America	Mass vaccination centre	393 (17.8)	393 (30.3)
		B	90%	No booster needed	1/1,000,000	China	Your local pharmacy	521 (23.6)	521 (40.1)
	6	A	80%	Booster every year	Unknown risk	China	Your local pharmacy	151 (6.8)	151 (11.6)
		B	100%	No booster needed	1/10,000	European Union	Mass vaccination centre	843 (38.2)	843 (64.9)
	7	A	100%	Booster every 6 months	1/100,000	European Union	Your general practice	857 (38.8)	857 (66)
		B	80%	Booster every year	1/10,000	United States of America	Mass vaccination centre	163 (7.4)	163 (12.6)
	8	A	80%	Booster every 6 months	1/1,000,000	European Union	Your local pharmacy	784 (35.5)	784 (60.4)

		B	50%	Booster every year	1/100,000	United States of America	Your general practice	213 (9.7)	213 (16.4)
Block B (n = 2208)	1	A	90%	No booster needed	1/100,000	United States of America	Mass vaccination centre	622 (28.2)	622 (48.1)
		B	50%	Booster every year	1/10,000	European Union	Your general practice	350 (15.9)	350 (27)
	2	A	80%	Booster every 6 months	1/10,000	China	Your general practice	202 (9.1)	202 (15.6)
		B	100%	Booster every year	Unknown risk	European Union	Mass vaccination centre	627 (28.4)	627 (48.5)
	3	A	100%	Booster every 6 months	1/1,000,000	United States of America	Your general practice	778 (35.2)	778 (60.1)
		B	50%	No booster needed	1/10,000	China	Your local pharmacy	154 (7)	154 (11.9)
	4	A	50%	No booster needed	1/100,000	European Union	Your local pharmacy	334 (15.1)	334 (25.8)
		B	90%	Booster every year	1/1,000,000	United States of America	Your local pharmacy	660 (29.9)	660 (51)
	5	A	100%	No booster needed	Unknown risk	China	Mass vaccination centre	235 (10.6)	235 (18.2)
		B	90%	Booster every 6 months	1/100,000	United States of America	Your general practice	612 (27.7)	612 (47.3)
	6	A	100%	Booster every 6 months	1/10,000	China	Your local pharmacy	235 (10.6)	235 (18.2)
		B	80%	No booster needed	1/1,000,000	European Union	Mass vaccination centre	835 (37.8)	835 (64.5)
	7	A	50%	No booster needed	1/1,000,000	United States of America	Your local pharmacy	530 (24)	530 (41)
		B	90%	Booster every 6 months	Unknown risk	China	Mass vaccination centre	272 (12.3)	272 (21)
	8	A	50%	Booster every 6 months	Unknown risk	United States of America	Mass vaccination centre	207 (9.4)	207 (16)
		B	80%	Booster every year	1/100,000	China	Your general practice	547 (24.8)	547 (42.3)

Note: Refusal of both vaccines A and B is deducted from 1-uptake of vaccines A or B in each choice task.

Table S2. Prevalence of detected SARS-CoV-2 infection in the French working age population.

	Survey experiment (from 30 November to 16 December 2020)		Epi model estimates (as of 15 January 2021)
	n **(%)**	**Prevalence, % (95% CI)**	**Prevalence, % (95% CI)**
**Metropolitan France**	6007 (100)	8.1 (7.5–8.8)	7.8 (6.0–10.0)
Region of residency (ordered by SARS-CoV-2 prevalence)			
Normandie (NOR)	306 (5.1)	4.2 (2.3–7.2)	4.4 (3.4–5.7)
Bretagne (BRE)	301 (5.0)	5.0 (2.8–8.1)	2.6 (2.1–3.5)
Centre-Val de Loire (CVL)	234 (3.9)	5.1 (2.7–8.8)	5.4 (4.2–7.0)
Pays-de-la-Loire (PDL)	344 (5.7)	5.2 (3.1–8.1)	4.1 (3.1–5.3)
Nouvelle-Aquitaine (NAQ)	541 (9.0)	6.3 (4.4–8.7)	3.1 (2.4–4.0)
Bourgogne-Franche-Comté (BFC)	256 (4.3)	7.4 (4.5–11.3)	8.2 (6.4–10.8)
Grand Est (GES)	527 (8.8)	7.6 (5.5–10.2)	9.1 (7.1–11.9)
Occitanie (OCC)	534 (8.9)	8.1 (5.9–10.7)	4.4 (3.5–5.8)
Provence-Alpes-Côte-d'Azur (PAC)	456 (7.6)	9.0 (6.5–12.0)	10.7 (8.3–14.1)
Hauts-de-France (HDF)	569 (9.5)	9.1 (6.9–11.8)	7.3 (5.7–9.6)
Auvergne-Rhône-Alpes (ARA)	741 (12.3)	9.4 (7.4–11.8)	7.7 (6.0–10.1)
Ile-de-France (IDF)	1198 (19.9)	10.9 (9.2–12.8)	13.2 (10.3–17.2)
Correlation coefficient (*p*-value)		0.81 (0.002)	

Note: Epi model estimates were retrieved for the working age population (20–64 years) from Hozé et al. Monitoring the proportion of the population infected by SARS-CoV-2 using age-stratified hospitalisation and serological data: a modelling study. *The Lancet Public health* 2021, 6, e408-e415.

Table S3. Characteristics of participants without prior SARS-CoV-2 infection by region of residency (December 2020, France).

Characteristics	French region of residency (ordered by SARS-CoV-2 prevalence)												*p*-value
	NOR	BRE	CVL	PDL	NAQ	BFC	GES	OCC	PAC	HDF	ARA	IDF	
N (%)	231 (5.2)	228 (5.2)	175 (4.0)	261 (5.9)	409 (9.3)	190 (4.3)	400 (9.1)	393 (8.9)	327 (7.4)	418 (9.5)	547 (12.4)	836 (18.9)	
Survey stratification variables (by region of residency)													
Gender													0.70
Women	129 (55.8)	119 (52.2)	93 (53.1)	140 (53.6)	226 (55.3)	95 (50.0)	220 (55.0)	216 (55.0)	186 (56.9)	211 (50.5)	278 (50.8)	460 (55.0)	
Men	102 (44.2)	109 (47.8)	82 (46.9)	121 (46.4)	183 (44.7)	95 (50.0)	180 (45.0)	177 (45.0)	141 (43.1)	207 (49.5)	269 (49.2)	376 (45.0)	
Age group													0.016
18–24 years	18 (7.8)	23 (10.1)	8 (4.6)	32 (12.3)	33 (8.1)	21 (11.1)	39 (9.8)	35 (8.9)	30 (9.2)	60 (14.4)	72 (13.2)	67 (8.0)	
25–34 years	40 (17.3)	37 (16.2)	34 (19.4)	42 (16.1)	56 (13.7)	27 (14.2)	65 (16.3)	70 (17.8)	48 (14.7)	73 (17.5)	91 (16.6)	181 (21.7)	
35–44 years	49 (21.2)	51 (22.4)	42 (24.0)	54 (20.7)	104 (25.4)	44 (23.2)	92 (23.0)	90 (22.9)	72 (22.0)	88 (21.1)	119 (21.8)	219 (26.2)	
45–54 years	58 (25.1)	62 (27.2)	41 (23.4)	69 (26.4)	105 (25.7)	50 (26.3)	109 (27.3)	95 (24.2)	92 (28.1)	103 (24.6)	134 (24.5)	205 (24.5)	
55–64 years	66 (28.6)	55 (24.1)	50 (28.6)	64 (24.5)	111 (27.1)	48 (25.3)	95 (23.8)	103 (26.2)	85 (26.0)	94 (22.5)	131 (23.9)	164 (19.6)	
Educational achievement													<0.001
Some high school	128 (55.4)	90 (39.5)	92 (52.6)	123 (47.1)	201 (49.1)	90 (47.4)	203 (50.8)	177 (45.0)	162 (49.5)	210 (50.2)	245 (44.8)	298 (35.6)	
High school graduate	47 (20.3)	63 (27.6)	36 (20.6)	59 (22.6)	95 (23.2)	41 (21.6)	98 (24.5)	98 (24.9)	72 (22.0)	95 (22.7)	114 (20.8)	183 (21.9)	
University graduate	56 (24.2)	75 (32.9)	47 (26.9)	79 (30.3)	113 (27.6)	59 (31.1)	99 (24.8)	118 (30.0)	93 (28.4)	113 (27.0)	188 (34.4)	355 (42.5)	
Household size													
1 adult	63 (27.3)	67 (29.4)	44 (25.1)	63 (24.1)	111 (27.1)	43 (22.6)	105 (26.3)	106 (27.0)	78 (23.9)	79 (18.9)	127 (23.2)	206 (24.6)	
2 adults	131 (56.7)	127 (55.7)	105 (60.0)	157 (60.2)	228 (55.7)	111 (58.4)	236 (59.0)	231 (58.8)	188 (57.5)	259 (62.0)	322 (58.9)	475 (56.8)	0.47
≥3 adults	37 (16.0)	34 (14.9)	26 (14.9)	41 (15.7)	70 (17.1)	36 (18.9)	59 (14.8)	56 (14.2)	61 (18.7)	80 (19.1)	98 (17.9)	155 (18.5)	
No child	139 (60.2)	141 (61.8)	100 (57.1)	161 (61.7)	244 (59.7)	113 (59.5)	232 (58.0)	233 (59.3)	201 (61.5)	231 (55.3)	316 (57.8)	498 (59.6)	
1 child	39 (16.9)	36 (15.8)	29 (16.6)	39 (14.9)	83 (20.3)	41 (21.6)	82 (20.5)	83 (21.1)	60 (18.3)	82 (19.6)	110 (20.1)	169 (20.2)	0.85
2 children	41 (17.7)	33 (14.5)	29 (16.6)	43 (16.5)	61 (14.9)	26 (13.7)	58 (14.5)	52 (13.2)	50 (15.3)	72 (17.2)	89 (16.3)	123 (14.7)	
≥3 children	12 (5.2)	18 (7.9)	17 (9.7)	18 (6.9)	21 (5.1)	10 (5.3)	28 (7.0)	25 (6.4)	16 (4.9)	33 (7.9)	32 (5.9)	46 (5.5)	
Area of residence													<0.001
Rural area	75 (32.5)	63 (27.6)	56 (32.0)	64 (24.5)	119 (29.1)	82 (43.2)	103 (25.8)	86 (21.9)	16 (4.9)	100 (23.9)	116 (21.2)	37 (4.4)	

Urban area <100,000 inhabitants	87 (37.7)	113 (49.6)	70 (40.0)	106 (40.6)	137 (33.5)	68 (35.8)	153 (38.3)	161 (41.0)	66 (20.2)	134 (32.1)	178 (32.5)	56 (6.7)	
Urban area ≥100,000 inhabitants	69 (29.9)	52 (22.8)	49 (28.0)	91 (34.9)	153 (37.4)	40 (21.1)	144 (36.0)	146 (37.2)	245 (74.9)	184 (44.0)	253 (46.3)	743 (88.9)	
Variables related to vaccination behavior													
Working status													<0.001
Healthcare worker	16 (6.9)	22 (9.6)	16 (9.1)	22 (8.4)	43 (10.5)	20 (10.5)	38 (9.5)	38 (9.7)	33 (10.1)	31 (7.4)	42 (7.7)	62 (7.4)	
Worker in contact with the public	75 (32.5)	61 (26.8)	44 (25.1)	65 (24.9)	143 (35.0)	58 (30.5)	108 (27.0)	108 (27.5)	114 (34.9)	98 (23.4)	157 (28.7)	281 (33.6)	
Worker not in contact with the public	61 (26.4)	64 (28.1)	55 (31.4)	96 (36.8)	94 (23.0)	44 (23.2)	112 (28.0)	99 (25.2)	81 (24.8)	110 (26.3)	164 (30.0)	284 (34.0)	
Not working	79 (34.2)	81 (35.5)	60 (34.3)	78 (29.9)	129 (31.5)	68 (35.8)	142 (35.5)	148 (37.7)	99 (30.3)	179 (42.8)	184 (33.6)	209 (25.0)	
Unemployed or furloughed because of the COVID-19 health crisis													0.022
Yes	34 (14.7)	34 (14.9)	27 (15.4)	57 (21.8)	65 (15.9)	32 (16.8)	75 (18.8)	73 (18.6)	62 (19.0)	61 (14.6)	92 (16.8)	186 (22.2)	
Not reported	197 (85.3)	194 (85.1)	148 (84.6)	204 (78.2)	344 (84.1)	158 (83.2)	325 (81.3)	320 (81.4)	265 (81.0)	357 (85.4)	455 (83.2)	650 (77.8)	
Compliance with recommended vaccinations in the past													0.003
Always	114 (49.4)	113 (49.6)	78 (44.6)	120 (46.0)	185 (45.2)	90 (47.4)	166 (41.5)	152 (38.7)	110 (33.6)	178 (42.6)	233 (42.6)	375 (44.9)	
Sometimes	83 (35.9)	87 (38.2)	70 (40.0)	108 (41.4)	170 (41.6)	75 (39.5)	184 (46.0)	174 (44.3)	144 (44.0)	189 (45.2)	238 (43.5)	353 (42.2)	
Never	34 (14.7)	28 (12.3)	27 (15.4)	33 (12.6)	54 (13.2)	25 (13.2)	50 (12.5)	67 (17.0)	73 (22.3)	51 (12.2)	76 (13.9)	108 (12.9)	
Vaccination against influenza 2020/2021													0.084
Yes	40 (17.3)	37 (16.2)	33 (18.9)	29 (11.1)	63 (15.4)	34 (17.9)	70 (17.5)	48 (12.2)	42 (12.8)	56 (13.4)	65 (11.9)	121 (14.5)	
Not reported	191 (82.7)	191 (83.8)	142 (81.1)	232 (88.9)	346 (84.6)	156 (82.1)	330 (82.5)	345 (87.8)	285 (87.2)	362 (86.6)	482 (88.1)	715 (85.5)	
At-risk group of severe COVID-19													
Pregnancy (among women)													0.17
Yes	0 (0.0)	1 (0.8)	3 (3.2)	1 (0.7)	4 (1.8)	0 (0.0)	1 (0.5)	4 (1.9)	2 (1.1)	3 (1.4)	10 (3.6)	10 (2.2)	
Not reported	129 (100)	118 (99.2)	90 (96.8)	139 (99.3)	222 (98.2)	95 (100)	219 (99.6)	212 (98.2)	184 (98.9)	208 (98.6)	268 (96.4)	450 (97.3)	
Smoking status													0.37
Current smoker	73 (31.6)	58 (25.4)	46 (26.3)	58 (22.2)	108 (26.4)	48 (25.3)	115 (28.8)	108 (27.5)	88 (26.9)	114 (27.3)	152 (27.8)	211 (25.2)	
Former smoker	80 (34.6)	90 (39.5)	58 (33.1)	99 (37.9)	151 (36.9)	58 (30.5)	130 (32.5)	135 (34.4)	117 (35.8)	136 (32.5)	175 (32.0)	261 (31.2)	
Never smoker	78 (33.8)	80 (35.1)	71 (40.6)	104 (39.8)	150 (36.7)	84 (44.2)	155 (38.8)	150 (38.2)	122 (37.3)	168 (40.2)	220 (40.2)	364 (43.5)	
Body mass index													0.016

Obesity (≥30 kg/m2)	54 (23.4)	43 (18.9)	34 (19.4)	37 (14.2)	73 (17.8)	28 (14.7)	106 (26.5)	74 (18.8)	53 (16.2)	88 (21.1)	97 (17.7)	149 (17.8)	
Overweight (25–30 kg/m2)	73 (31.6)	82 (36.0)	66 (37.7)	82 (31.4)	138 (33.7)	63 (33.2)	121 (30.3)	127 (32.3)	102 (31.2)	142 (34.0)	164 (30.0)	276 (33.0)	
Normal weight (≤25 kg/m2)	104 (45.0)	103 (45.2)	75 (42.9)	142 (54.4)	198 (48.4)	99 (52.1)	173 (43.3)	192 (48.9)	172 (52.6)	188 (45.0)	286 (52.3)	411 (49.2)	
Hypertension													<0.001
Yes	18 (7.8)	23 (10.1)	14 (8.0)	27 (10.3)	34 (8.3)	15 (7.9)	67 (16.8)	27 (6.9)	20 (6.1)	51 (12.2)	42 (7.7)	73 (8.7)	
Not reported	213 (92.2)	205 (89.9)	161 (92.0)	234 (89.7)	375 (91.7)	175 (92.1)	333 (83.3)	366 (93.1)	307 (93.9)	367 (87.8)	505 (92.3)	763 (91.3)	
Diabetes mellitus													0.24
Yes	14 (6.1)	9 (3.9)	8 (4.6)	7 (2.7)	19 (4.6)	9 (4.7)	24 (6.0)	15 (3.8)	14 (4.3)	27 (6.5)	18 (3.3)	29 (3.5)	
Not reported	217 (93.9)	219 (96.1)	167 (95.4)	254 (97.3)	390 (95.4)	181 (95.3)	376 (94.0)	378 (96.2)	313 (95.7)	391 (93.5)	529 (96.7)	807 (96.5)	
Other chronic condition*													0.068
Yes	28 (12.1)	23 (10.1)	18 (10.3)	22 (8.4)	35 (8.6)	16 (8.4)	50 (12.5)	30 (7.6)	24 (7.3)	48 (11.5)	43 (7.9)	61 (7.3)	
Not reported	203 (87.9)	205 (89.9)	157 (89.7)	239 (91.6)	374 (91.4)	174 (91.6)	350 (87.5)	363 (92.4)	303 (92.7)	370 (88.5)	504 (92.1)	775 (92.7)	
Experience of COVID-19													
Had COVID-19 symptoms (without medical confirmation)													<0.001
Yes	25 (10.8)	31 (13.6)	26 (14.9)	36 (13.8)	54 (13.2)	21 (11.1)	90 (22.5)	79 (20.1)	76 (23.2)	74 (17.7)	114 (20.8)	202 (24.2)	
Not reported	206 (89.2)	197 (86.4)	149 (85.1)	225 (86.2)	355 (86.8)	169 (88.9)	310 (77.5)	314 (79.9)	251 (76.8)	344 (82.3)	433 (79.2)	634 (75.8)	
Had a test for SARS-CoV-2 infection diagnosis (with negative result)													0.19
Yes	36 (15.6)	34 (14.9)	35 (20.0)	39 (14.9)	69 (16.9)	34 (17.9)	65 (16.3)	74 (18.8)	77 (23.5)	79 (18.9)	94 (17.2)	166 (19.9)	
Not reported	195 (84.4)	194 (85.1)	140 (80.0)	222 (85.1)	340 (83.1)	156 (82.1)	335 (83.8)	319 (81.2)	250 (76.5)	339 (81.1)	453 (82.8)	670 (80.1)	
Knows someone who got COVID-19													<0.001
Yes with hospital admission	32 (13.9)	26 (11.4)	30 (17.1)	35 (13.4)	42 (10.3)	38 (20.0)	72 (18.0)	67 (17.0)	66 (20.2)	60 (14.4)	126 (23.0)	186 (22.2)	
Yes without hospital admission	80 (34.6)	88 (38.6)	65 (37.1)	121 (46.4)	162 (39.6)	92 (48.4)	168 (42.0)	161 (41.0)	152 (46.5)	184 (44.0)	261 (47.7)	397 (47.5)	
Not reported	119 (51.5)	114 (50.0)	80 (45.7)	105 (40.2)	205 (50.1)	60 (31.6)	160 (40.0)	165 (42.0)	109 (33.3)	174 (41.6)	160 (29.3)	253 (30.3)	
Curfew in the municipality													<0.001

Starting on 17 October 2020	89 (38.5)	0 (0.0)	0 (0.0)	0 (0.0)	0 (0.0)	0 (0.0)	0 (0.0)	185 (47.1)	117 (35.8)	180 (43.1)	300 (54.8)	836 (100.0)	
Starting on 28 October 2020	51 (22.1)	75 (32.9)	92 (52.6)	64 (24.5)	66 (16.1)	92 (48.4)	242 (60.5)	159 (40.5)	199 (60.9)	158 (37.8)	211 (38.6)	0 (0.0)	
Starting on 15 December 2020	91 (39.4)	153 (67.1)	83 (47.4)	197 (75.5)	343 (83.9)	98 (51.6)	158 (39.5)	49 (12.5)	11 (3.4)	80 (19.1)	36 (6.6)	0 (0.0)	
Perceived severity of COVID-19 if infected													0.002
Very severe	20 (8.7)	24 (10.5)	15 (8.6)	13 (5.0)	17 (4.2)	17 (8.9)	34 (8.5)	23 (5.9)	18 (5.5)	32 (7.7)	29 (5.3)	65 (7.8)	
Somewhat severe	79 (34.2)	64 (28.1)	55 (31.4)	84 (32.2)	152 (37.2)	60 (31.6)	138 (34.5)	124 (31.6)	99 (30.3)	168 (40.2)	165 (30.2)	303 (36.2)	
Not particularly severe	75 (32.5)	84 (36.8)	53 (30.3)	90 (34.5)	148 (36.2)	62 (32.6)	128 (32.0)	129 (32.8)	121 (37.0)	117 (28.0)	213 (38.9)	276 (33.0)	
Not severe at all	15 (6.5)	25 (11.0)	9 (5.1)	28 (10.7)	26 (6.4)	23 (12.1)	38 (9.5)	40 (10.2)	34 (10.4)	32 (7.7)	55 (10.1)	67 (8.0)	
Don't know	42 (18.2)	31 (13.6)	43 (24.6)	46 (17.6)	66 (16.1)	28 (14.7)	62 (15.5)	77 (19.6)	55 (16.8)	69 (16.5)	85 (15.5)	125 (15.0)	
Background information on COVID-19 vaccination													
Herd immunity against SARS-CoV-2													0.099
>66% of adults aged 18–64 years old must be immunized (either by vaccination or infection)	83 (35.9)	75 (32.9)	64 (36.6)	86 (33.0)	138 (33.7)	70 (36.8)	130 (32.5)	136 (34.6)	118 (36.1)	124 (29.7)	184 (33.6)	264 (31.6)	
>50% of adults aged 18–64 years old must be immunized (either by vaccination or infection)	85 (36.8)	69 (30.3)	52 (29.7)	84 (32.2)	145 (35.5)	40 (21.1)	143 (35.8)	124 (31.6)	108 (33.0)	159 (38.0)	180 (32.9)	282 (33.7)	
No information	63 (27.3)	84 (36.8)	59 (33.7)	91 (34.9)	126 (30.8)	80 (42.1)	127 (31.8)	133 (33.8)	101 (30.9)	135 (32.3)	183 (33.5)	290 (34.7)	
General practitioner's advice on vaccination													0.47
Recommendation	111 (48.1)	108 (47.4)	99 (56.6)	133 (51.0)	194 (47.4)	92 (48.4)	212 (53.0)	184 (46.8)	157 (48.0)	221 (52.9)	277 (50.6)	427 (51.1)	
No opinion	120 (51.9)	120 (52.6)	76 (43.4)	128 (49.0)	215 (52.6)	98 (51.6)	188 (47.0)	209 (53.2)	170 (52.0)	197 (47.1)	270 (49.4)	409 (48.9)	

* Normandie (NOR); Bretagne (BRE) ; Centre-Val de Loire (CVL) ; Pays-de-la-Loire (PDL) ; Nouvelle-Aquitaine (NAQ) ; Bourgogne-Franche-Comté (BFC) ; Grand Est (GES) ; Occitanie (OCC) ; Provence-Alpes-Côte d'Azur (PAC) ; Hauts-de-France (HDF) ; Auvergne-Rhône-Alpes (ARA) ; Ile-de-France (IDF).
** Chronic conditions include: 137 (34%) asthma; 130 (33%) chronic lung disease other than asthma; 35 (9%) chronic arterial disease; 95 (24%) chronic heart disease; 18 (5%) chronic kidney disease; 39 (10%) cancer.

Table S4. Outright refusal of COVID-19 vaccination (December 2020, France).

Characteristics	All participants without SARS-CoV-2 infection	Outright refusal of COVID-19 vaccination		*p*-value
		Yes	No	
***N* (%)**	**4415 (100.0)**	**1823 (41.3)**	2592 (58.7)	
French region of residency (ordered by SARS-CoV-2 prevalence)				0.18
Normandie (NOR)	231 (5.2)	95 (41.1)	136 (58.9)	
Bretagne (BRE)	228 (5.2)	85 (37.3)	143 (62.7)	
Centre-Val de Loire (CVL)	175 (4.0)	70 (40.0)	105 (60.0)	
Pays-de-la-Loire (PDL)	261 (5.9)	97 (37.2)	164 (62.8)	
Nouvelle-Aquitaine (NAQ)	409 (9.3)	183 (44.7)	226 (55.3)	
Bourgogne-Franche-Comté (BFC)	190 (4.3)	76 (40.0)	114 (60.0)	
Grand Est (GES)	400 (9.1)	159 (39.8)	241 (60.3)	
Occitanie (OCC)	393 (8.9)	185 (47.1)	208 (52.9)	
Provence-Alpes-Côte-d'Azur (PAC)	327 (7.4)	144 (44.0)	183 (56.0)	
Hauts-de-France (HDF)	418 (9.5)	170 (40.7)	248 (59.3)	
Auvergne-Rhône-Alpes (ARA)	547 (12.4)	234 (42.8)	313 (57.2)	
Ile-de-France (IDF)	836 (18.9)	325 (38.9)	511 (61.1)	
Survey stratification variables (by region of residency)				
Gender				<0.001
Women	2373 (53.7)	1139 (48.0)	1234 (52.0)	
Men	2042 (46.3)	684 (33.5)	1358 (66.5)	
Age group				<0.001
18–24 years	438 (9.9)	153 (34.9)	285 (65.1)	
25–34 years	764 (17.3)	359 (47.0)	405 (53.0)	
35–44 years	1024 (23.2)	446 (43.6)	578 (56.4)	
45–54 years	1123 (25.4)	465 (41.4)	658 (58.6)	
55–64 years	1066 (24.1)	400 (37.5)	666 (62.5)	
Educational achievement				<0.001
Some high school	2019 (45.7)	929 (46.0)	1090 (54.0)	
High school graduate	1001 (22.7)	423 (42.3)	578 (57.7)	
University graduate	1395 (31.6)	471 (33.8)	924 (66.2)	
Household size				
1 adult	1092 (24.7)	458 (41.9)	634 (58.1)	
2 adults	2570 (58.2)	1075 (41.8)	1495 (58.2)	0.24
≥3 adults	753 (17.1)	290 (38.5)	463 (61.5)	
No child	2609 (59.1)	1028 (39.4)	1581 (60.6)	
1 child	853 (19.3)	375 (44.0)	478 (56.0)	0.006
2 children	677 (15.3)	286 (42.2)	391 (57.8)	
≥3 children	276 (6.3)	134 (48.6)	142 (51.4)	
Area of residence				<0.001
Rural area	917 (20.8)	413 (45.0)	504 (55.0)	
Urban area <100,000 inhabitants	1329 (30.1)	578 (43.5)	751 (56.5)	
Urban area ≥100,000 inhabitants	2169 (49.1)	832 (38.4)	1337 (61.6)	
Variables related to vaccination behavior				

Working status				0.95
Healthcare worker	383 (8.7)	160 (41.8)	223 (58.2)	
Worker in contact with the public	1312 (29.7)	533 (40.6)	779 (59.4)	
Worker not in contact with the public	1264 (28.6)	523 (41.4)	741 (58.6)	
Not working	1456 (33.0)	607 (41.7)	849 (58.3)	
Unemployed or furloughed because of the COVID-19 health crisis				0.66
Yes	798 (18.1)	324 (40.6)	474 (59.4)	
Not reported	3617 (81.9)	1499 (41.4)	2118 (58.6)	
Compliance with recommended vaccinations in the past				<0.001
Always	1914 (43.4)	515 (26.9)	1399 (73.1)	
Sometimes	1875 (42.5)	870 (46.4)	1005 (53.6)	
Never	626 (14.2)	438 (70.0)	188 (30.0)	
Vaccination against influenza 2020/2021				<0.001
Yes	638 (14.5)	128 (20.1)	510 (79.9)	
Not reported	3777 (85.5)	1695 (44.9)	2082 (55.1)	
At-risk group of severe COVID-19				
Pregnancy (among women)				0.024
Yes	39 (1.6)	23 (59.0)	16 (41.0)	
Not reported	2334 (98.4)	1116 (47.8)	1218 (52.2)	
Smoking status				0.38
Current smoker	1179 (26.7)	507 (43.0)	672 (57.0)	
Former smoker	1490 (33.7)	607 (40.7)	883 (59.3)	
Never smoker	1746 (39.5)	709 (40.6)	1037 (59.4)	
Body mass index				0.54
Obesity (≥30 kg/m2)	836 (18.9)	332 (39.7)	504 (60.3)	
Overweight (25-30 kg/m2)	1436 (32.5)	604 (42.1)	832 (57.9)	
Normal weight (≤25 kg/m2)	2143 (48.5)	887 (41.4)	1256 (58.6)	
Hypertension				<0.001
Yes	411 (9.3)	123 (29.9)	288 (70.1)	
Not reported	4004 (90.7)	1700 (42.5)	2304 (57.5)	
Diabetes mellitus				<0.001
Yes	193 (4.4)	54 (28.0)	139 (72.0)	
Not reported	4222 (95.6)	1769 (41.9)	2453 (58.1)	
Other chronic condition*				<0.001
Yes	398 (9.0)	126 (31.7)	272 (68.3)	
Not reported	4017 (91.0)	1697 (42.2)	2320 (57.8)	
Experience of COVID-19				
Had COVID-19 symptoms (without medical confirmation)				0.14
Yes	828 (18.8)	323 (39.0)	505 (61.0)	
Not reported	3587 (81.2)	1500 (41.8)	2087 (58.2)	
Had a test for SARS-CoV-2 infection diagnosis (with negative result)				0.038
Yes	802 (18.2)	305 (38.0)	497 (62.0)	
Not reported	3613 (81.8)	1518 (42.0)	2095 (58.0)	
Knows someone who got COVID-19				<0.001

Yes with hospital admission	780 (17.7)	291 (37.3)	489 (62.7)	
Yes without hospital admission	1931 (43.7)	759 (39.3)	1172 (60.7)	
Not reported	1704 (38.6)	773 (45.4)	931 (54.6)	
Curfew in the municipality				0.46
Starting on 17 October 2020	1707 (38.7)	685 (40.1)	1022 (59.9)	
Starting on 28 October 2020	1409 (31.9)	591 (41.9)	818 (58.1)	
Starting on 15 December 2020	1299 (29.4)	547 (42.1)	752 (57.9)	
Perceived severity of COVID-19 if infected				<0.001
Very severe	307 (7.0)	89 (29.0)	218 (71.0)	
Somewhat severe	1491 (33.8)	530 (35.5)	961 (64.5)	
Not particularly severe	1496 (33.9)	648 (43.3)	848 (56.7)	
Not severe at all	392 (8.9)	205 (52.3)	187 (47.7)	
Don't know	729 (16.5)	351 (48.1)	378 (51.9)	
Background information on COVID-19 vaccination				
Herd immunity against SARS-CoV-2				0.34
>66% of adults aged 18–64 years old must be immunized (either by vaccination or infection)	1472 (33.3)	588 (39.9)	884 (60.1)	
>50% of adults aged 18–64 years old must be immunized (either by vaccination or infection)	1471 (33.3)	608 (41.3)	863 (58.7)	
No information	1472 (33.3)	627 (42.6)	845 (57.4)	
General practitioner's advice on vaccination				0.78
Recommendation	2215 (50.2)	910 (41.1)	1305 (58.9)	
No opinion	2200 (49.8)	913 (41.5)	1287 (58.5)	

* Chronic conditions include: 137 (34%) asthma; 130 (33%) chronic lung disease other than asthma; 35 (9%) chronic arterial disease; 95 (24%) chronic heart disease; 18 (5%) chronic kidney disease; 39 (10%) cancer.

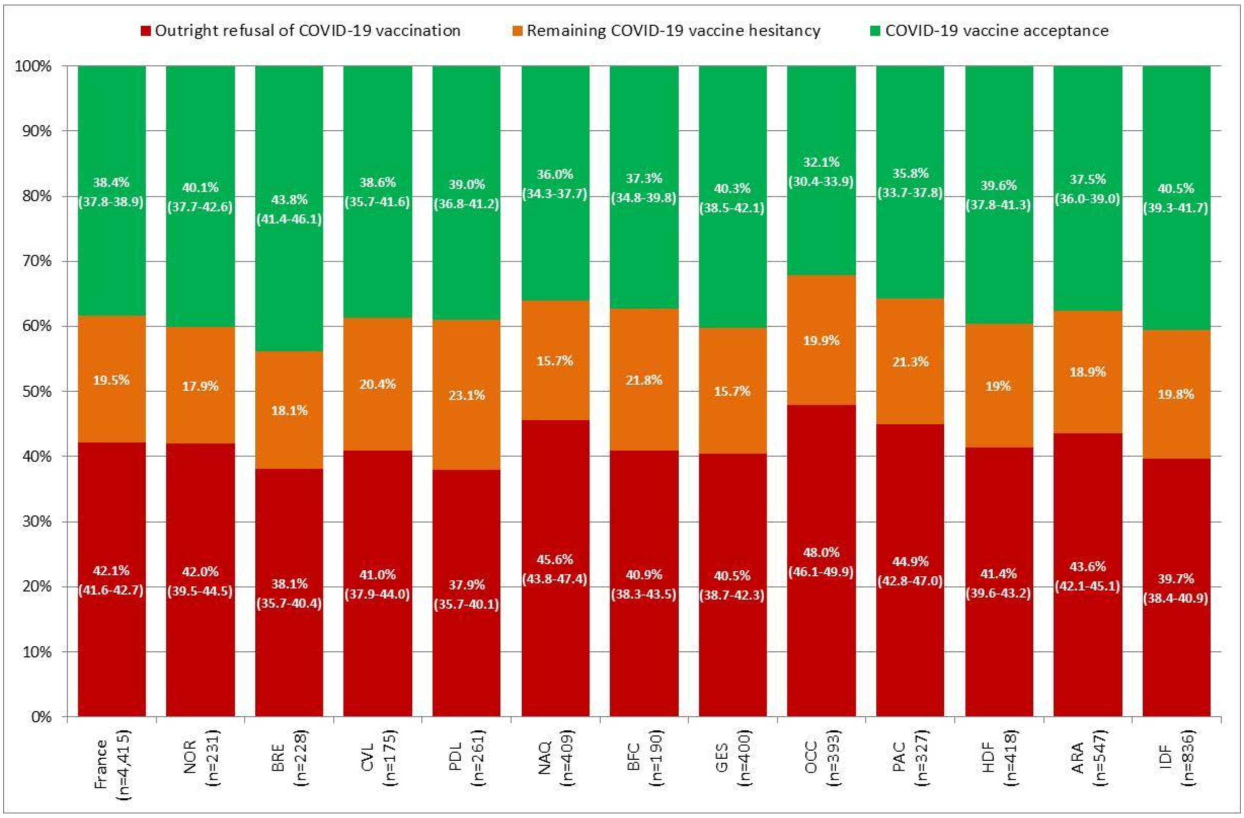

Figure S1. Acceptance of Pfizer or Moderna vaccines predicted in the working age population (December 2020, France).

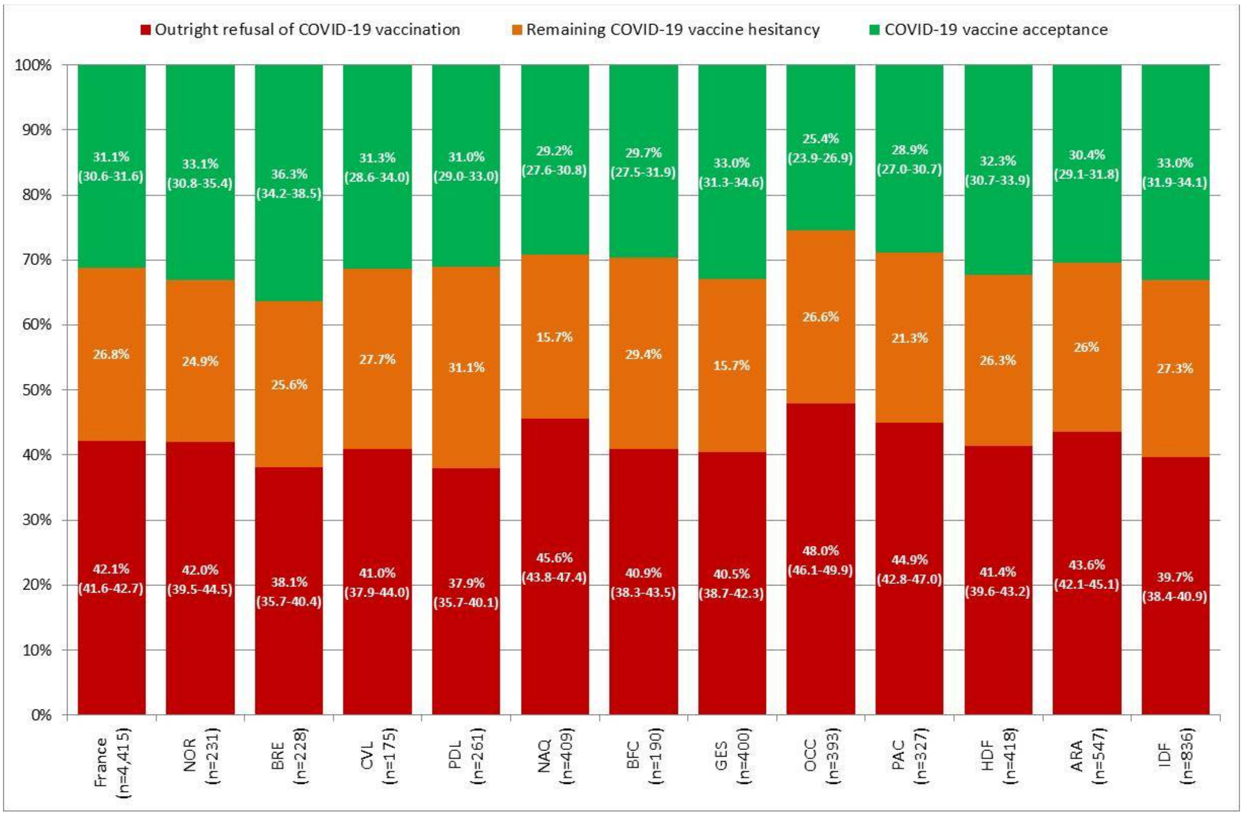

Figure S2. Acceptance of AstraZeneca vaccine predicted in the working age population (December 2020, France).
